# Association between household income and mental health among adults during the COVID-19 pandemic in Korea: Insights from a community health survey

**DOI:** 10.1371/journal.pone.0289230

**Published:** 2024-04-11

**Authors:** Min Hui Moon, Min Hyeok Choi

**Affiliations:** 1 Department of Preventive and Occupational & Environmental Medicine, Medical College, Pusan National University, Yangsan, Republic of Korea; 2 Office of Public Healthcare Service, Pusan National University Yangsan Hospital, Yangsan, Republic of Korea; Ajou University School of Medicine and Graduate School of Medicine, REPUBLIC OF KOREA

## Abstract

People of low socioeconomic status are vulnerable to health problems during disasters such as the COVID-19 pandemic. Using data from the 2019 and 2021 Korea Community Health Survey, this study analyzed the associations between Korean adults’ mental health and their national and regional-level household incomes during the pandemic. The prevalence of perceived stress and depression experience for each risk factor category was calculated through univariate analyses. A multivariate logistic regression analysis helped identify the association between two types of income levels (national or regional) and perceived stress and experience of depression. Additionally, we investigated the effect of income levels by subgroup (gender and residential area) on perceived stress and the experience of depression. During the pandemic, the crude prevalence of an experience of depression was higher (6.24% to 7.2%) but that of perceived stress remained unchanged. Regarding regional-income based mental health disparities, even after adjusting for each independent variable, perceived stress (2019 odds ratio (OR): 1.26, 95% confidence interval (CI):1.26–1.27, 2021 OR: 1.32, 95% CI: 1.32–1.32) and experience of depression (2019 OR: 1.56, 95% CI: 1.55–1.56, 2021 OR: 1.63, 95% CI: 1.63–1.64) increased as the income level decreased. The perceived stress based on the two income levels was higher in women than in men. For both income levels, the experience of depression of women was higher than that of men before COVID-19 and vice versa during the COVID-19 period. National income had a more pronounced effect on mental health in urban areas than in rural areas. Contrarily, the effect of regional income level on mental health was not consistent across residential areas (urban and rural areas). Our findings demonstrated that mental health disparities based on income level were more likely to occur during the COVID-19 pandemic and are better reflected through disparities in regional income levels.

## Introduction

The coronavirus disease (COVID-19) pandemic has significantly impacted mental health worldwide, including stress, anxiety, and depression [1, 2]. According to a 2021 Organization for Economic Cooperation and Development (OECD) report, the prevalence of depression in OECD countries has approximately doubled since the COVID-19 pandemic, with the prevalence being the highest in Korea at 36.8% among OECD countries [3]. During the COVID-19 pandemic, social distancing and containment measures had a direct impact on mental health [4–6]. Several studies have reported that large-scale infectious diseases can cause emotional confusion and difficulties such as depression and anxiety [7–10]. Cao et al. [7] and Shevlin et al. [10] explored the effects of the COVID-19 pandemic and social isolation on mental health, and Lee et al. [9] investigated factors related to fear of the COVID-19 infection and its psychological and social impact. Furthermore, studies have shown that social isolation due to COVID-19 and fear and awareness of the infection increases symptoms of depression and anxiety [4].

Mental health related to stress and depression is affected by socioeconomic risk factors such as education level, occupation, and income level; mental health in low-income groups is particularly aggravated by poverty and income inequality [11, 12]. According to Patel et al.’s [13] review study on the effect of income level on mental health disparities among representative socioeconomic factors, 33 surveys conducted in 20 countries reported that the lower the income level, the higher the risk of depression. Hong et al. [14] found that inequality based on income level is more pronounced in mental health than in physical health, doubling the size of inequality over 10 years. Several other studies have consistently shown that people with lower socioeconomic status are more vulnerable to mental health problems [13–17]. Many countries have conducted studies on regional inequalities, particularly in health status between urban and rural areas [18–22]. Research on health inequality between urban and rural areas seeks ways to improve health disparities among regions by analyzing factors such as medical accessibility, living environment, socioeconomic factors, and cultural differences among regions. Korea has a significant disparity in income levels and access to medical services between urban and rural areas. Several studies have identified mental health disparities based on income level at the national level. Meanwhile, Song and Kim [15] studied income inequality at the national as well as regional levels. As income inequality at the regional level has a significant impact on social capital and access to healthcare infrastructure, measuring health gaps is more useful than measuring income inequality at the national level [15, 16].

The COVID-19 pandemic has intensified health disparities among individuals with low socioeconomic status. Specifically, individuals with lower levels of education, income, or unstable employment face a higher risk of COVID-19 infection, leading to increased severity of the disease and higher mortality rates [23–27]. Hall et al. [23] examined the impact of income inequality on daily life and mental health during the COVID-19 pandemic. The results showed that the low-income population had difficulty purchasing food and daily necessities, and their health status deteriorated because of a lack of time and resources for proper healthcare. Owing to this influence, the low-income group showed unstable mental health conditions, such as stress and depression, compared to the high-income group.

Previous studies have confirmed that a) mental health status, in terms of stress and depression, deteriorated owing to the COVID-19 pandemic, and b) there were differences in mental health based on income level [13–15]. These studies were often limited to analyzing the effect of income inequality at the national level; few studies have analyzed the relationship between income inequality and mental health status at the regional level, which can reflect the accessibility of healthcare infrastructure in the region.

Therefore, this study aimed to identify the relationships between people’s mental health and their national and regional-level household incomes during COVID-19 pandemic. In addition, the study aimed to provide a policy direction for improving mental health disparities by comparing the patterns of health inequality based on national and regional income levels.

Therefore, the purpose of this study was to:

1. Analyze the relationship between mental health (perceived stress and the experience of depression) based on the income levels of the total (national household income level) and regional (regional household income level) population during the COVID-19 pandemic.
2. Investigate the effect of income levels by subgroup (gender and residential area) on perceived stress and experiences of depression.

## Materials and methods

### Data and study population

Data were obtained from the Korea Community Health Survey (KCHS). Since 2008, the Community Health Survey (CHS) has been conducted annually by the Korea Centers for Disease Control and Prevention. The CHS is a large-scale survey in which approximately 220,000 people nationwide participate from August to October every year and includes questions on chronic disease screening, health behavior, food intake, and socioeconomic status. Survey data on health level, health behavior, food and nutrition intake, and chronic disease prevalence in Korea were used as official national indicators (Korea Community Health Survey Guidelines, website: http://chs.kdca.go.kr/). This survey was conducted in the form of a 1:1 interview with a surveyor visiting households of adults aged 19 or older residing in 255 cities, counties, and districts in Korea. Research participants were selected through probability proportional sampling and systematic sampling each year. For this study, raw data was requested from the Korea Centers for Disease Control and Prevention and approved on March 15, 2023. The KCHS data for 2019 and 2021 consists of a total of 458,341 individuals. In this study, the final study population comprised 449,234 (223,288 in 2019 and 225,946 in 2021) individuals, excluding 9,107 (5,811 in 2019 and 3,296 in 2021) individuals with missing information.

### Variables

#### Dependent variables

The dependent variables included perceived stress and experience of depression; measures for both variables are based on widely accepted standards and are employed in various research studies [15, 28, 29]. Perceived stress and depression are representative indicators of mental health, and stress plays an important role in predicting depression [30–32]. Several studies have demonstrated that exposure to perceived stress and experiences of depression are associated with poor health outcomes and affect socioeconomic imbalance [33–35]. Perceived stress was assessed using the question, “How stressful do you feel in your daily life?” with response options of “feel very much,” “feel a lot,” “feel a little bit,” and “hardly feel it.”. For the analysis, those who responded “I feel it very much” and “I feel it a lot” were classified as those who usually feel stress in my daily life, and those who answered “I feel it a little” and “I hardly feel it” was classified as a people who do not feel stressed in daily life. The experience of depression was surveyed using the following question: “Did you feel sadness or despair enough to bother you in your daily life for more than 2 weeks in the last year?” Their answer was recorded as “yes” or “no.” In this study, a participant was defined as one who answered “yes.”

#### Independent variables

The variable for the socioeconomic status, considered a major factor in this study, was the income level. The income level is an indicator of socioeconomic status that can be used to directly measure the material resources available to individuals. Income level is closely related to health; the lower the income level, the higher the rate of unhealthy states [36, 37]. The income level used in this study was household equalized income. As the number of household members varies from household to household, it is important to calculate household equivalization income by considering the household equivalence index. The household equalized income is the sum of the household’s total annual income divided by the square root of the number of household members. The income level in whole country (Korea) is classified into tertiles based on equalized household income. The income level of the region was calculated by dividing the household equivalized income into tertiles for each of the 255 cities and their respective counties and districts.

The general factors associated with perceived stress and experience of depression were included as independent variables after reviewing studies that previously reported mental health risk factors [38–40]. Demographic variables included gender (men or women), age group (19–34 years, 35–64 years, or ≥65 years), and area of residence (urban or rural). Social-economic parameters included education level (≤middle school, ≤high school, or college or above), job status (economic activity or non-economic activity), marital status (married or not married) and the basic livelihood condition. Health behavior factors included current smoking (yes or no), high-risk drinking (men: drinking seven standard drinks or more over once a week, women: drinking five standard drinks or more over once a week), and walking practice (walking activity for ≥ 30 min, ≥ five days in the previous week).

### Statistical analysis

Univariate analysis was performed to reveal the general characteristics of all variables used in the study. Statistically significant differences between each independent variable and the mental health status (perceived stress and experiences of depression) were verified using the Rao-Scott chi-square test. The association between the two types of income levels (national and regional income levels) and mental health (perceived stress and experience of depression) were analyzed using complex-sample multivariate logistic regression while adjusting for the confounding variables. Subgroup analysis was performed based on two types of income levels (national and regional income levels), gender, and area of residence. Statistical significance was set at a p-value of <0.05. All statistical analyses were performed using SAS 9.4 (SAS Institute, Cary, NC, USA).

### Ethical considerations

This study was approved by the Institutional Review Board (IRB) of Pusan National University Hospital (IRB No. 04-2022-030). All the participants provided written informed consent for the KCHS. The survey was conducted after sufficiently explaining to the participants that the results would be used for statistical purposes only and that confidentiality was guaranteed. The need for informed consent was waived off by the IRB because the data were analyzed anonymously.

## Results

Table 1 shows the general characteristics of the study population. The total number of participants was 449,234, and the number of weighted analysis participants was 84,491,967 (41,590,294 in 2019 and 42,901,673 in 2021). The proportions of men and women were similar (49.66% in 2019 and 49.62% in 2021), and those aged 35–64 years were the most common subgroup population (56.79% in 2019 and 55.67% in 2021). Regarding population distribution according to the Korean national income levels, the proportion of individuals with a high income level was the highest, followed by the proportions of individuals with a middle income level and those with a low income level. The same was found for regional income levels. The prevalence of perceived stress was 24.78% in both 2019 and 2021. The prevalence of experience of depression was 6.25% and 7.2% in 2019 and 2021, respectively.

**Table 1:** General characteristics of the study population.

		2019			2021		
		N	Weight N	Weight %	N	Weight N	Weight %
**Total**		223,288	41,590,294	100.00	225,946	42,901,673	100.00
**Gender**	**Men**	100,076	20,653,435	49.66	103,022	21,287,731	49.62
	**Women**	123,212	20,936,859	50.34	122,924	21,613,942	50.38
**Age group**	**19–34**	33,539	10,057,727	24.18	35,622	10,167,904	23.70
	**35–64**	117,048	23,618,138	56.79	116,791	23,882,188	55.67
	**≥65**	72,701	7,914,429	19.03	73,533	8,851,581	20.63
**Area of residence**	**Urban**	124,739	33,750,880	81.15	127,315	34,870,498	81.28
	**Rural**	98,549	7,839,414	18.85	98,631	8,031,175	18.72
**Education**	**Middle school**	79,077	8,303,254	19.96	72,123	7,962,562	18.56
	**High school**	63,962	12,329,258	29.64	65,490	12,488,195	29.11
	**College and above**	80,249	20,957,782	50.39	88,333	22,450,916	52.33
**Job status**	**Economically active**	138,739	26,473,047	63.65	141,544	27,324,490	63.69
	**Non-active**	84,549	15,117,247	36.35	84,402	15,577,183	36.31
**Marital status**	**Married**	148,579	26,627,752	64.02	142,120	26,190,336	61.05
	**Not married**	74,709	14,962,542	35.98	83,826	16,711,337	38.95
**National income level** ^a^	**High**	75,415	18,462,860	44.39	80,798	19,667,569	45.84
	**Middle**	74,948	14,451,252	34.75	70,232	13,493,676	31.45
	**Low**	72,925	8,676,182	20.86	74,916	9,740,428	22.70
**Regional income level** ^a^	**High**	76,829	15,797,359	37.98	77,732	16,008,586	37.31
	**Middle**	71,527	13,580,193	32.65	72,386	13,889,434	32.38
	**Low**	74,932	12,212,742	29.36	75,828	13,003,653	30.31
**Basic livelihood condition**	**Yes**	8,837	1,312,334	3.16	10,692	1,694,212	3.95
	**No**	214,451	40,277,960	96.84	215,254	41,207,461	96.05
**Smoking status**	**Yes**	186,250	33,694,788	81.02	189,588	35,342,643	82.38
	**No**	37,038	7,895,506	18.98	36,358	7,559,030	17.62
**High-risk drinking** ^b^	**Yes**	198,249	36,136,004	86.89	205,720	38,690,084	90.18
	**No**	25,039	5,454,290	13.11	20,226	4,211,589	9.82
**Physical activity** ^c^	**Yes**	89,671	18,967,814	45.61	93,778	19,377,491	45.17
	**No**	133,617	22,622,480	54.39	132,168	23,524,182	54.83
**Perceived stress**	**Yes**	49,319	10,305,831	24.78	50,169	10,630,372	24.78
	**No**	173,969	31,284,463	75.22	175,777	32,271,301	75.22
**Experience of depression**	**Yes**	13,731	2,598,126	6.25	16,129	3,088,224	7.20
	**No**	209,557	38,992,168	93.75	209,817	39,813,449	92.80

^a^Categorized using equivalent income

^b^Drinking alcohol: men who drank seven standard drinks or more once a week; women who drank five standard drinks or more once a week

^c^Walking activity ≥ 30 minutes, ≥ five days in the last week

Table 2 shows perceived stress and experiences of depression according to the factors identified in before the COVID-19 (2019) and during the COVID-19 (2021). Perceived stress was significantly higher in women than in men in both 2019 and 2021 (p < 0.0001). It was most common in the 19–34-year-old population subgroup, followed by among those aged 35–64 and ≥65 years. Both before and during the COVID-19 period, the prevalence of perceived stress based on income level in Korea was significantly higher at higher income levels. Before COVID-19, the prevalence of perceived stress by regional income level was the highest in the high regional-income-level, followed by the low regional-income-level and middle regional-income-level groups. During COVID-19, the prevalence of perceived stress by regional income level was significantly higher at higher income levels. The number of people living in urban areas was higher than in rural areas. Economic activity was significantly higher than in the non-economic activity group (p < 0.0001). Depression was significantly higher in women than in men both before and during the COVID-19 and occurred mostly in the ≥65 years age group. The experience of depression based on the both national and regional income levels was significantly higher at lower income levels.

**Table 2:** Perceived stress and experience of depression according to factors during the COVID-19 pandemic.

		Perceived stress				Experience of depression			
		2019		2021		2019		2021	
		N	%	N	%	N	%	N	%
**Gender**	**Men**	20,789	20.77	21,384	20.76	4,350	4.35	5,315	5.16
	**Women**	28,530	23.16	28,785	23.42	9,381	7.61	10,814	8.80
***p*-value** ^a^		<0.0001		<0.0001		<0.0001		<0.0001	
**Age group**	**19–34**	9,591	28.60	10,042	28.19	1,835	5.47	2,246	6.31
	**35–64**	27,691	23.66	28,834	24.69	6,762	5.78	8,078	6.92
	**≥65**	12,037	16.56	11,293	15.36	5,134	7.06	5,805	7.89
***p*-value**		<0.0001		<0.0001		<0.0001		<0.0001	
**Area of residence**	**Urban**	29,908	23.98	30,809	24.20	8,002	6.41	9,513	7.47
	**Rural**	19,411	19.70	19,360	19.63	5,729	5.81	6,616	6.71
***p*-value**		<0.0001		<0.0001		<0.0001		<0.0001	
**Education**	**Middle school**	14,977	18.94	12,720	17.64	6,071	7.68	6,431	8.92
	**High school**	14,005	21.90	14,807	22.61	3,859	6.03	4,613	7.04
	**College and above**	20,337	25.34	22,642	25.63	3,801	4.74	5,085	5.76
***p*-value**		<0.0001		<0.0001		<0.0001		<0.0001	
**Job status**	**Economically active**	32,579	23.48	33,707	23.81	6,539	4.71	8,034	5.68
	**Non-active**	16,740	19.80	16,462	19.50	7,192	8.51	8,095	9.59
***p*-value**		<0.0001		<0.0001		<0.0001		<0.0001	
**Marital status**	**Married**	31,855	21.44	30,680	21.59	7,679	5.17	8,507	5.99
	**Not married**	17,464	23.38	19,489	23.25	6,052	8.1	7,622	9.09
***p*-value**		<0.0001		<0.0001		<0.0001		<0.0001	
**National income level**	**High**	17,713	23.49	19,217	23.78	3,328	4.41	4,217	5.22
	**Middle**	16,244	21.67	15,456	22.01	4,071	5.43	4,381	6.24
	**Low**	15,362	21.07	15,496	20.68	6,332	8.68	7,531	10.05
***p*-value**		<0.0001		<0.0001		<0.0001		<0.0001	
**Regional Income level**	**High**	17,237	22.44	17,670	22.73	3,313	4.31	3,964	5.10
	**Middle**	15,551	21.74	16,074	22.21	3,786	5.29	4,523	6.25
	**Low**	16,531	22.06	16,425	21.66	6,632	8.85	7,642	10.08
***p*-value**		<0.0001		<0.0001		<0.0001		<0.0001	
**Basic livelihood condition**	**Yes**	2,761	31.24	3,132	29.29	1,443	16.33	1,930	18.05
	**No**	46,558	21.71	47,037	21.85	12,288	5.73	14,199	6.60
***p*-value**		<0.0001		<0.0001		<0.0001		<0.0001	
**Smoking status**	**Yes**	10,406	28.10	10,288	28.30	2,391	6.46	2,776	7.64
	**No**	38,913	20.89	39,881	21.04	11,340	6.09	13,353	7.04
***p*-value**		<0.0001		<0.0001		<0.0001		<0.0001	
**High-risk drinking**	**Yes**	6,887	27.51	5,745	28.40	1,418	5.66	1,439	7.11
	**No**	42,432	21.40	44,424	21.59	12,313	6.21	14,690	7.14
***p*-value**		<0.0001		<0.0001		<0.0001		<0.0001	
**Physical activity**	**Yes**	18,727	20.88	19,411	20.70	4,914	5.48	5,947	6.34
	**No**	30,592	22.90	30,758	23.27	8,817	6.60	10,182	7.70
***p*-value**		<0.0001		<0.0001		<0.0001		<0.0001	

^a^ Results of the Rao-Scott chi-square test

Table 3 presents the influence of the two types of income levels (national and regional income levels) on perceived stress and experiences of depression by gender. Both types of income levels were significant factors affecting perceived stress and depression after adjusting for the impacts of other factors in before (2019) and during (2021) the COVID-19 period. The odds ratio (OR) of national income level of perceived stress was 1.31 (95% confidence interval (CI) 1.30–1.31, p < 0.0001) and 1.30 (95% CI 1.29–1.30, p < 0.0001) in 2019 and 2021, respectively. Considering the regional income level, the perceived stress for the low-income level group was 1.26 (95% CI 1.26–1.27) and 1.32 (95% CI 1.32–1.32) in 2019 and 2021, respectively. Compared to before the COVID-19 pandemic, perceived stress according to income level was lower at the national income level and higher at the regional income level during the COVID-19 pandemic. The experience of depression for both income levels was higher during the pandemic than before the pandemic. The prevalence of perceived stress by the two income level was found to be higher in women than in men. The prevalence of experience of depression was higher in women than in men before the COVID-19 pandemic for both income types; this trend was reversed during the pandemic.

**Table 3:** Influence of the national and regional income levels on perceived stress and experience of depression by gender based on complex-sample multivariate logistic regression analysis.

		Perceived stress				Experience of depression			
		2019		2021		2019		2021	
		Adj OR (95% CI)	*p*-value	Adj OR (95% CI)	*p*-value	Adj OR (95% CI)	*p*-value	Adj OR (95% CI)	*p*-value
**Total** ^a^									
**National income level**	**High**	Reference		Reference		Reference		Reference	
	**Middle**	1.09 (1.09–1.09)	<0.0001	1.10 (1.10–1.11)	<0.0001	1.21 (1.21–1.22)	<0.0001	1.17 (1.17–1.18)	<0.0001
	**Low**	1.31 (1.30–1.31)	<0.0001	1.30 (1.29–1.30)	<0.0001	1.72 (1.72–1.73)	<0.0001	1.73 (1.72–1.73)	<0.0001
**Regional income level**	**High**	Reference		Reference		Reference		Reference	
	**Middle**	1.07 (1.07–1.07)	<0.0001	1.11 (1.11–1.11)	<0.0001	1.12 (1.11–1.12)	<0.0001	1.11 (1.11–1.12)	<0.0001
	**Low**	1.26 (1.26–1.27)	<0.0001	1.32 (1.32–1.32)	<0.0001	1.56 (1.55–1.56)	<0.0001	1.63 (1.63–1.64)	<0.0001
**Men** ^b^									
**National income level**	**High**	Reference		Reference		Reference		Reference	
	**Middle**	1.04 (1.04–1.04)	<0.0001	1.08 (1.08–1.09)	<0.0001	1.15 (1.14–1.15)	<0.0001	1.13 (1.12–1.13)	<0.0001
	**Low**	1.22 (1.22–1.22)	<0.0001	1.25 (1.25–1.25)	<0.0001	1.65 (1.64–1.66)	<0.0001	1.75 (1.74–1.76)	<0.0001
**Regional income level**	**High**	Reference		Reference		Reference		Reference	
	**Middle**	1.05 (1.04–1.05)	<0.0001	1.08 (1.08–1.08)	<0.0001	1.12 (1.12–1.13)	<0.0001	1.08 (1.07–1.08)	<0.0001
	**Low**	1.21 (1.21–1.21)	<0.0001	1.28 (1.27–1.28)	<0.0001	1.49 (1.48–1.50)	<0.0001	1.65 (1.64–1.66)	<0.0001
**Women** ^b^									
**National income level**	**High**	Reference		Reference		Reference		Reference	
	**Middle**	1.14 (1.14–1.14)	<0.0001	1.13 (1.12–1.13)	<0.0001	1.24 (1.24–1.25)	<0.0001	1.19 (1.19–1.20)	<0.0001
	**Low**	1.38 (1.38–1.39)	<0.0001	1.33 (1.33–1.34)	<0.0001	1.72 (1.71–1.72)	<0.0001	1.68 (1.67–1.68)	<0.0001
**Regional income level**	**High**	Reference		Reference		Reference		Reference	
	**Middle**	1.09 (1.09–1.10)	<0.0001	1.14 (1.14–1.14)	<0.0001	1.11 (1.11–1.12)	<0.0001	1.13 (1.13–1.14)	<0.0001
	**Low**	1.31 (1.31–1.32)	<0.0001	1.36 (1.36–1.36)	<0.0001	1.57 (1.56–1.57)	<0.0001	1.59 (1.58–1.60)	<0.0001

^a^ Adj OR, adjusted for gender, age, area of residence, education, job status, marital status, basic livelihood condition, smoking status, high-risk drinking, and physical activity

^b^ Adj OR, adjusted for age, area of residence, education, job status, marital status, basic livelihood condition, smoking status, high-risk drinking, and physical activity. Adj OR, adjusted odds ratio; CI, confidence interval.

Table 4 shows the results of the analysis of the influence of the two income levels on mental health (perceived stress and experience of depression) by subgroup (gender and residential area). Excluding the national income level of men living in rural areas in 2021, both the national and regional income levels were significantly associated with mental health in all analyses (p < 0.0001). The influence of the national income level on mental health (perceived stress and experience of depression) was found to be greater in urban areas than in rural areas. Conversely, the influence of the regional income level on mental health (perceived stress and experience of depression) was inconsistent across areas of residence (urban and rural). During the COVID-19 period, the magnitude of mental health disparities based on the national income level was higher in rural areas than in urban areas; conversely, the extent of mental health disparities based on the regional income level was either higher or remained unchanged across urban and rural areas. The magnitude of disparity in perceived stress by gender was higher for women in both before (2019) and during (2021) the COVID-19 period. The magnitude inequality in experience of depression was greater for women than for men in before COVID-19 (2019); however, during the COVID-19 (2021) period, the degree of inequality was greater for men than for women.

**Table 4:** Influence of the national and regional income levels on perceived stress and experience of depression by gender and area of residence based on complex-sample multivariate logistic regression analysis.

			Perceived stress				Experience of depression			
			2019		2021		2019		2021	
			Adj OR (95% CI)	*p*-value	Adj OR (95% CI)	*p*-value	Adj OR (95% CI)	*p*-value	Adj OR (95% CI)	*p*-value
**Total** ^a^										
**Urban**	**National income level**	**High**	Reference		Reference		Reference		Reference	
		**Middle**	1.11 (1.11–1.11)	<0.0001	1.13 (1.12–1.13)	<0.0001	1.23 (1.23–1.23)	<0.0001	1.17 (1.17–1.18)	<0.0001
		**Low**	1.34 (1.33–1.34)	<0.0001	1.31 (1.31–1.32)	<0.0001	1.76 (1.75–1.77)	<0.0001	1.73 (1.73–1.74)	<0.0001
	**Regional income level**	**High**	Reference		Reference		Reference		Reference	
		**Middle**	1.07 (1.07–1.07)	<0.0001	1.12 (1.12–1.12)	<0.0001	1.10 (1.10–1.11)	<0.0001	1.10 (1.10–1.10)	<0.0001
		**Low**	1.26 (1.26–1.27)	<0.0001	1.31 (1.31–1.32)	<0.0001	1.54 (1.53–1.55)	<0.0001	1.63 (1.63–1.64)	<0.0001
**Rural**	**National income level**	**High**	Reference		Reference		Reference		Reference	
		**Middle**	0.98 (0.98–0.99)	<0.0001	1.01 (1.00–1.01)	0.0004	1.12 (1.11–1.13)	<0.0001	1.17 (1.16–1.18)	<0.0001
		**Low**	1.16 (1.15–1.16)	<0.0001	1.22 (1.21–1.22)	<0.0001	1.53 (1.51–1.54)	<0.0001	1.71 (1.70–1.73)	<0.0001
	**Regional income level**	**High**	Reference		Reference		Reference		Reference	
		**Middle**	1.08 (1.07–1.08)	<0.0001	1.07 (1.06–1.07)	<0.0001	1.19 (1.18–1.20)	<0.0001	1.18 (1.17–1.19)	<0.0001
		**Low**	1.25 (1.25–1.26)	<0.0001	1.35 (1.34–1.35)	<0.0001	1.63 (1.62–1.65)	<0.0001	1.63 (1.62–1.64)	<0.0001
**Men** ^b^										
**Urban**	**National income level**	**High**	Reference		Reference		Reference		Reference	
		**Middle**	1.06 (1.06–1.06)	<0.0001	1.10 (1.10–1.10)	<0.0001	1.18 (1.17–1.18)	<0.0001	1.15 (1.15–1.16)	<0.0001
		**Low**	1.25 (1.25–1.26)	<0.0001	1.27 (1.26–1.27)	<0.0001	1.72 (1.71–1.74)	<0.0001	1.72 (1.71–1.73)	<0.0001
	**Regional income level**	**High**	Reference		Reference		Reference		Reference	
		**Middle**	1.04 (1.04–1.05)	<0.0001	1.08 (1.08–1.09)	<0.0001	1.12 (1.11–1.13)	<0.0001	1.07 (1.06–1.07)	<0.0001
		**Low**	1.22 (1.21–1.22)	<0.0001	1.26 (1.26–1.27)	<0.0001	1.48 (1.47–1.49)	<0.0001	1.66 (1.65–1.67)	<0.0001
**Rural**	**National income level**	**High**	Reference		Reference		Reference		Reference	
		**Middle**	0.94 (0.93–0.94)	<0.0001	1.00 (0.99–1.00)	0.1135	1.00 (0.99–1.02)	0.8016	1.01 (1.00–1.02)	0.2529
		**Low**	1.08 (1.08–1.09)	<0.0001	1.18 (1.17–1.19)	<0.0001	1.36 (1.34–1.38)	<0.0001	1.80 (1.77–1.82)	<0.0001
	**Regional income level**	**High**	Reference		Reference		Reference		Reference	
		**Middle**	1.07 (1.06–1.07)	<0.0001	1.05 (1.04–1.05)	<0.0001	1.13 (1.12–1.15)	<0.0001	1.12 (1.11–1.14)	<0.0001
		**Low**	1.15 (1.15–1.16)	<0.0001	1.33 (1.32–1.34)	<0.0001	1.50 (1.48–1.52)	<0.0001	1.58 (1.56–1.60)	<0.0001
**Women** ^b^										
**Urban**	**National income level**	**High**	Reference		Reference		Reference		Reference	
		**Middle**	1.16 (1.16–1.16)	<0.0001	1.15 (1.14–1.15)	<0.0001	1.25 (1.25–1.26)	<0.0001	1.17 (1.17–1.18)	<0.0001
		**Low**	1.41 (1.41–1.42)	<0.0001	1.34 (1.34–1.35)	<0.0001	1.74 (1.73–1.75)	<0.0001	1.69 (1.69–1.70)	<0.0001
	**Regional income level**	**High**	Reference		Reference		Reference		Reference	
		**Middle**	1.09 (1.09–1.10)	<0.0001	1.15 (1.14–1.15)	<0.0001	1.09 (1.09–1.10)	<0.0001	1.12 (1.11–1.12)	<0.0001
		**Low**	1.31 (1.30–1.31)	<0.0001	1.35 (1.35–1.36)	<0.0001	1.54 (1.53–1.55)	<0.0001	1.58 (1.58–1.59)	<0.0001
**Rural**	**National income level**	**High**	Reference		Reference		Reference		Reference	
		**Middle**	1.04 (1.03–1.04)	<0.0001	1.03 (1.02–1.04)	<0.0001	1.19 (1.18–1.20)	<0.0001	1.28 (1.27–1.29)	<0.0001
		**Low**	1.23 (1.22–1.23)	<0.0001	1.27 (1.26–1.28)	<0.0001	1.59 (1.57–1.61)	<0.0001	1.65 (1.64–1.67)	<0.0001
	**Regional income level**	**High**	Reference		Reference		Reference		Reference	
		**Middle**	1.10 (1.09–1.10)	<0.0001	1.10 (1.09–1.11)	<0.0001	1.22 (1.21–1.24)	<0.0001	1.21 (1.20–1.23)	<0.0001
		**Low**	1.34 (1.33–1.35)	<0.0001	1.38 (1.37–1.39)	<0.0001	1.68 (1.67–1.70)	<0.0001	1.65 (1.64–1.67)	<0.0001

^a^ Adj OR, adjusted for gender, age, education, job status, marital status, basic livelihood condition, smoking status, high-risk drinking, and physical activity

^b^ Adj OR, adjusted for age, education, job status, marital status, basic livelihood condition, smoking status, high-risk drinking, and physical activity. Adj OR, adjusted odds ratio; CI, confidence interval.

## Discussion

To the best of our knowledge, this was the first study in Korea to compare mental health disparities based on income at the national and regional levels using the CHS before and during the COVID-19 period. This study intended to provide basic data necessary for policy development to resolve mental health disparities caused by income inequalities during large-scale infectious diseases.

During the COVID-19 pandemic, although the crude prevalence of experience of depression was higher than that before the pandemic, the crude prevalence of perceived stress remained similar. Regarding mental health disparities by income level, even after adjusting for each independent variable, perceived stress and the experience of depression increased as the income level decreased. Hall et al. [23] found that low-income groups had less access to resources for responding to COVID-19 and suffered more economic stress than high-income groups. These economic difficulties reportedly have a negative impact on daily life and mental health [23, 41, 42]. During the COVID-19 period, the perceived stress decreased based on the national income level and increased based on the regional income level; in contrast, for the same period, the experience of depression increased for both types of income levels. When measuring inequality by income level, few previous studies have identified mental health disparities at the regional income level. Moreover, in some cases, mental health disparity may be more affected by income inequality at the regional level than at the national level. Aneshensel CS and Sucoff CA [43] and Mair C et al. [44] found that the economic situation (income level), population composition, and characteristics of the residential environment in the area of residence have an effect on depression symptoms. This result is consistent with those of previous studies that used the income inequality at the regional level as a measure of health inequality and as a significant indicator for identifying the relationship between income and depressive symptoms [15, 16].

In this study, the perceived stress according to two income levels was found to be higher in women than in men. The experience of depression for both types of income levels was higher for women than for men before COVID-19 and vice versa during the COVID-19 period. The result that women’s mental health had greater disparities than that of men is consistent with the results observed by Almeida et al. [45]. Before COVID-19, the OR for experiencing depression was higher for women than for men at both types of income levels. However, men had a higher OR of experiencing depression than women during the COVID-19 pandemic. This suggests that employment-related probabilities make men more likely to report mental health disparities. Employment is an important factor that allows individuals to become economically independent and improve their quality of life. However, owing to COVID-19 pandemic, unemployment increased, working hours were shortened, and labor force participation decreased. Many people lost their jobs owing to the lockdown imposed during the pandemic, which disrupted their daily lives and negatively impacted their mental health [46, 47].

Additionally, this study confirmed the regional characteristics (urban and rural areas) related to changes in mental health disparities according to income levels during the COVID-19 pandemic. The effect of the national income level on the prevalence of perceived stress was more pronounced in urban areas than in rural areas. The prevalence of experience of depression based on the national income level was also higher in urban areas than in rural areas. Conversely, the size of mental health (perceived stress and experience of depression) disparities by the regional income level was not consistent between rural and urban areas. This lack of consistency in results has been reported in previous studies, as the impact on mental health disparities in urban and rural areas often conflicts [18–21]. In urban areas, social distancing is more strictly practiced because of the high number of infected people, which may increase mental health disparities. Furthermore, people’s mental health may be more vulnerable in rural areas because of the lack of information, access, and social support. These results indicate that the income level during the COVID-19 pandemic can be expected to have different effects on mental health depending on regional characteristics; however, additional research needs to be conducted in the future.

The limitations of this study are as follows. First, the perceived stress and experience of depression, which were used as outcome variables, are values that record responses in the form of self-report rather than medical diagnosis, which raises the possibility of bias. Additionally, we did not specify the time-period respondents should consider when measuring perceived stress, so it is possible that respondents may interpret this in very different ways. However, as we used the items that are most frequently used in national surveys, the possibility of comparison with other studies related to this topic is high. Second, direct changes in mental health disparities across the sub periods of the COVID-19 pandemic were not identified. In Korea, the COVID-19 pandemic has usually been divided into three periods: the initial epidemic, delta mutation, and omicron epidemic phase. It is necessary to identify the difference in mental health disparities according to the income inequality within the detailed epidemic periods because the response strategies differ depending on the size of the epidemic and the impact of the restrictions on socioeconomic activities, such as social distancing. In addition, future studies will need to address how inequality will change even after the end of COVID-19.

## Conclusions

In this study, we confirmed the pattern and size of mental health disparities based on two types of income levels (national and regional) during the COVID-19 pandemic. This study also considered the gender- and residential-area-based differences on mental health inequality at the two types of income levels. To alleviate mental health disparities, income inequality should be reduced, and mental health policies should be intensively implemented, especially for socioeconomically unequal population groups.
